# Loneliness as a Predictor of Depression in Adults 50+: Findings from the Health and Retirement Study

**DOI:** 10.1093/geroni/igaf122.3114

**Published:** 2025-12-31

**Authors:** Helena Polansky, Debra Dobbs, Hongdao Meng

**Affiliations:** University of South Florida, Tampa, Florida, United States; University of South Florida, Tampa, Florida, United States; University of South Florida, Tampa, Florida, United States

## Abstract

Loneliness is a public health concern that negatively impacts mental well-being among older adults. Understanding its role in depressive symptoms is crucial for developing effective interventions to enhance psychological health and quality of life in aging populations. This study uses a cross-sectional design to examine loneliness as a predictor of depressive symptoms among adults 50 years and older, utilizing a nationally representative sample from Wave 15 (2020) of the Health and Retirement Study (HRS). The analytic sample (N = 4,212) self-reported data on the 11-item UCLA Loneliness Scale and the 8-item CES-D Scale. Additional variables included demographic factors (age, gender, education), self-rated health, chronic conditions, and functional limitations (Activities of Daily Living [ADLs], Instrumental Activities of Daily Living [IADLs]). Spearman correlation and multiple linear regression analyses were employed to assess the relationship between loneliness and depressive symptoms while controlling for covariates. Results indicated a significant positive correlation between loneliness and depressive symptoms (r = 0.35, p < 0.01). Regression analyses revealed loneliness significantly predicted depressive symptoms (β = 0.11, p < 0.01). Other significant predictors included self-rated health (β = 0.35, p < 0.01), chronic conditions (β = 0.12, p < 0.01), functional limitations (ADLs β = 0.24, p < 0.01; IADLs β = 0.38, p < 0.01), younger age, and female gender. The model explained approximately 29% of the variance in depressive symptoms. The findings underscore loneliness as a critical modifiable risk factor for depression among older adults. Addressing loneliness through targeted interventions could substantially improve mental health outcomes and overall quality of life for older adults. Future research should explore longitudinal designs to establish causality and investigate mechanisms linking loneliness and mental health outcomes.

